# Around the Hospitals

**Published:** 1894-09-22

**Authors:** 


### Around the Hospitals.

[Notice to the Managers of Institutions.—Special space is now reserved for the insertion of notices of hospital meetings and festivals. The Editor requests that all notices may reach The Hospital Office, 428, Strand, at least one week before the date of each meeting.]

**North London Hospital for Consumption.**—The new out-patient's quarters in Fitzroy Square have been in working order long enough to report that the change of locality has proved beneficial in every way. The new central block at the hospital was opened in July by the Princess Christian. This addition admits of the reception of 35 patients. To support the augmented institution more funds will be required, yet the building expenses have not been fully met, £3,000 remaining owing to the bankers, and the furnishing and equipment of the new block will absorb about £300. Unlike the case of the majority of hospitals, funds arising from annual subscriptions appear to be the most satisfactory source of income at Hampstead. These during the past year have shown an increase, whilst donations decreased and legacies were actually nil. The festival dinner brought in £1,200, and the purses presented to the Princess Christian at the opening of the new buildings produced £174. A very marked diminution is shown in the direction of congregational collections of the district during the last few years, and it would seem that renewed interest could be awakened in this direction, and generally in the direction of local efforts, as it is evident by the record shown in the annual report that much has been done in former years which is not done now. In connection with the work of a consumption hospital, especially, a convalescent fund should prove of great service. The contributors to this branch of the hospital numbered but eight, whilst those on the list of the incurable fund number but two. Two pages of the report are devoted to the latter fund, and it is pointed out what a boon a regular weekly allowance to sufferers could be. The disimbursement of £1 only during the year, shows that there is scope for much development in this direction. During the year 1893, 378 in-patients and 2,894 out-patients were treated. The ordinary income amounted to £3,368, and the ordinary expenditure to £5,411.

**The Miller Hospital, Greenwich.**—The district in which the Miller Hospital is situated is a poor and growing one, consequently the work of the hospital becomes more useful and more arduous year by year. The committee hopeto receive sufficient funds to enable them to erect two additional wards and a separate casualty department, the last being especially demanded owing to the large amount of accident cases calling for aid during the year. The hospital is forced through circumstances to acquire more land, it being necessary to purchase some adjoining stables to remove the disadvantage of their proximity. This purchase will entail an entrenchment on the invested funds of the hospital. We look for the refunding of this money, and the contribution of other sums towards new premises, from the neighbourhood, which does not fail to show its interest in the institution. During the year the licensed victuallers of Deptford and Lewisham contributed upwards of £95 to the hospital, whilst a local amateur dramatic society secured for it 100 guineas. The festival dinner, under the presidency of Sir Stuart Knill (the Lord Mayor), added £532 to the funds. Since the last annual meeting the management of the hospital have adopted the uniform system of accounts. During the year 203 in-patients and 9,915 out-patients have been attended.

